# Roman Republican coarse ware from *Norba*, Southern Lazio (Italy): a multi-analytical study of production technology and trade

**DOI:** 10.1007/s12520-023-01883-5

**Published:** 2023-11-06

**Authors:** Barbara Borgers, Corina Ionescu, Ágnes Gál, Tymon De Haas, Lucian Barbu-Tudoran

**Affiliations:** 1 Department of Classical Archaeology, University of Vienna, Franz Klein-Gasse 1, 1190 Vienna, Austria; 2 Electron Microscopy Center, Babeş-Bolyai University, 5-7 Clinicilor Str, 400006 Cluj-Napoca, Romania; 3 Department of Geology, Babeş-Bolyai University, 1 Kogălniceanu Str, 400084 Cluj-Napoca, Romania; 4 Institute of Archaeology, University of Groningen, Groningen, the Netherlands; 5 National Institute for Isotopic and Molecular Technologies (INCDTIM Cluj-Napoca), 67-103 Donath Str., Cluj-Napoca, Romania

**Keywords:** Roman Republican Coarse Ware, Archaeometric approach, Technology, Southern Lazio, Italy

## Abstract

The first objective of this paper is to reconstruct the production technology of fourth–first centuries BCE coarse ware from surveys near the ancient town of *Norba* in the Lepini Mountains of Southern Lazio, Italy, adopting a multi-analytical method, combining macroscopic observation with polarised light optical microscopy (OM), X-ray diffraction (XRD) and scanning electron microscopy (SEM). The second objective of this study is to gain insight into *Norba*’s integration in broader production and distribution networks in Southern Lazio between the fourth–first centuries BCE, by comparing the results with previous data for coarse ware prevalent in the region at that time. The results indicate that the coarse ware from *Norba* was produced with Fe-rich, Ca-poor, and illite-muscovite clays and fired in an oxidising atmosphere between 750 and 900 °C. Differences among the coarse ware exist in the paste recipes, e.g. intentionally added temper. Most coarse ware from *Norba* bears compositional similarities to that from the Alban Hills and the Tiber Valley, north of Rome, suggesting that *Norba* was integrated into the marketing of pottery that was common in Southern Lazio during the fourth–first centuries BCE. In comparison, only a few coarse wares seem to have been produced in the surrounding area (e.g. *Satricum* and *Forum Appii*), or even locally in *Norba*. The results further indicate changes in these regional/local distribution networks; some coarse ware seems to have been imported from *Satricum*, where a workshop was active during the fourth century BCE. When ceramic production at *Satricum* ceased, potters settled in the towns of *Forum Appii* and *Norba*, where they produced ceramic building material and fine ware in the second–first centuries BCE, respectively. The results of this study tentatively suggest that potters in these locations may have also manufactured coarse ware during this period.

## Introduction

Traditionally, studies on the Roman economy focus on the widespread distribution of specific pottery classes, and trace the movement of transport amphoras or black gloss fine ware, to map trade patterns (Horden and Purcell 2000). However, in order to gain insight into ancient sites’ specific positions within local and regional networks, analysis needs to be extended to other pottery classes, such as coarse ware (Launaro and Leone 2018), which, unlike amphoras and fine ware, usually constitutes the bulk of assemblages found on archaeological sites. Furthermore, studies of coarse ware have shown that they are mostly locally produced and distributed (Olcese 2003; Borgers et al. 2021). Consequently, coarse ware holds significant potential for tracing local and regional distribution and mapping trade networks.

By definition, the production of coarse ware requires coarse-grained materials in order to improve their thermal properties (Spataro and Villing 2015, and references therein). To this aim, non-plastic material, known as temper, can be added. For instance, the addition of coarse-grained quartz and K-feldspar is exceptionally well-suited for the manufacture of coarse ware—this long-standing practice has been identified in coarse ware produced at Vasanello in the Tiber River Valley, north of Rome, Central Italy (Peña 1992: 117).

Archaeometric studies of Roman Republican coarse ware in Central Italy have highlighted their potential in understanding aspects of production technology and regional trade patterns. More specifically, studies have indicated that coarse ware tends to be produced with Fe-rich and Ca-poor clays (Peña 1992; Olcese 2003). Production waste of coarse ware from a kiln at Tivoli, located east of Rome, is defined by the presence of coarse-grained leucite (Thierrin-Michael 2003: 58), while coarse ware from the workshop at *Satricum* located in the Pontine region, south of Rome, was manufactured with coarse-grained quartz and K-feldspar and accessory clinopyroxene (Attema et al. 2003).

Other archaeometric studies have focused on the reconstruction of regional trade networks of coarse ware from Rome, its suburbs, and its rural hinterland, and they indicated that three main productions circulated in the area during the Roman Republic and Early Imperial eras:

- Coarse ware with coarse-grained K-feldspar and quartz was distributed on various sites in Rome and Ostia (Schuring 1986, 1987; Thierrin-Michael 2003), as well as in the Pontine region, ca. 60 km south of Rome (Borgers et al. 2017);
- Coarse ware with coarse-grained leucite has been identified on settlement sites in Ostia (Capelli 2016: 196–198) and in Rome’s suburbs (Borgers and Fischetti 2023);
- Local coarse ware with coarse-grained rounded quartz, K-feldspar, and clinopyroxene from *Satricum* appears to have been distributed locally in the Pontine region (Borgers et al. 2017).

Following this, the present study adopts a multi-analytical approach for the examination of coarse ware from the Roman Republican era (fourth–first centuries BCE) found during surveys near ancient *Norba* (present-day Norma) on the foothills of the Lepini Mountains in Southern Lazio, Italy. This work has two main objectives: first, to reconstruct the production technology of the coarse ware, including raw materials (e.g. clay), paste recipes (e.g. temper), and firing processes (e.g. temperature and atmosphere). The second objective of this paper is to understand whether this part of Southern Lazio was integrated into the same production and trade networks of Rome, its suburbs, and its rural hinterland.

### Geological background

**Fig. 1:**
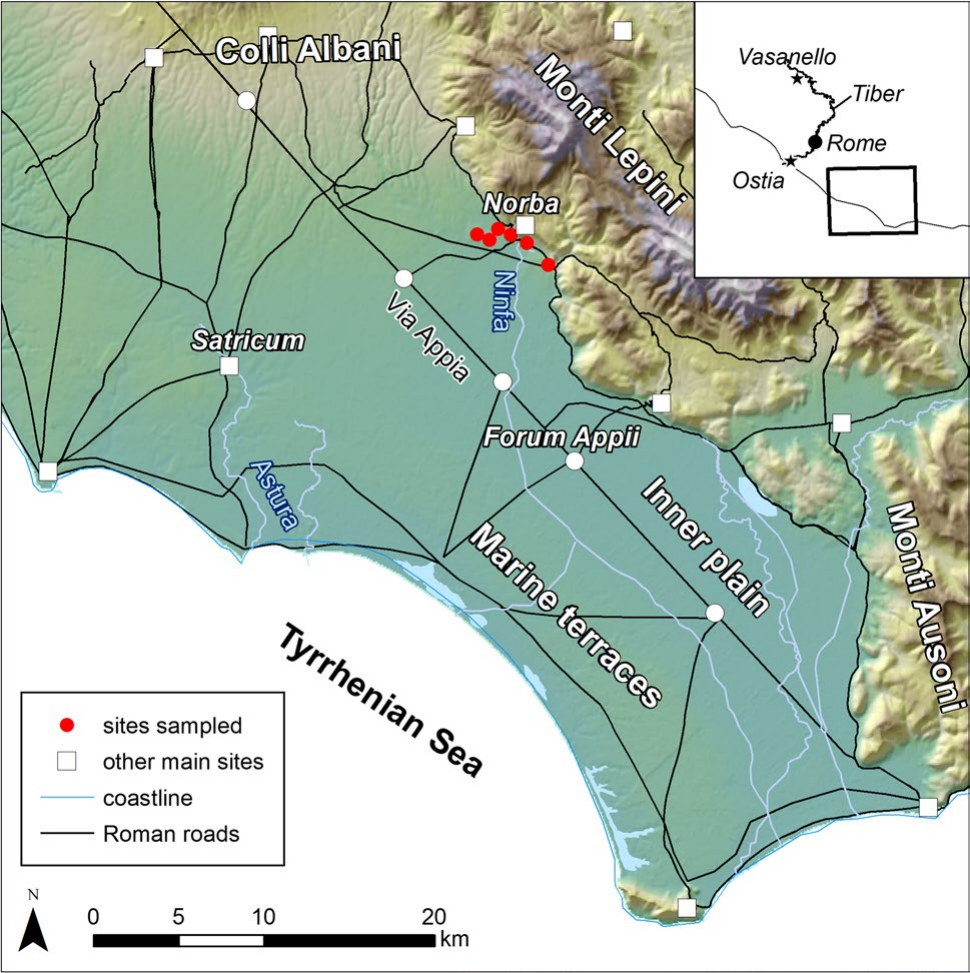
Location of the Pontine region in Southern Lazio, Italy, with the archaeological surveys (red dots) around *Norba* where the analysed coarse ware was found (Map compiled by T. De Haas)

The Pontine Region (Fig. 1) consists of a large coastal plain, comprising several marine terraces and an Inner plain (a graben), stretching from NW to SE, parallel with the sea coast. The graben of the Inner plain is filled in with Holocene peat and Fe-rich clayey sediments, with Fe- and Mn-rich nodules (Borgers et al. 2018; Sevink et al. 1984), and is crosscut by the Ninfa stream. To the north, the Pontine Region is bounded by the Alban Hills (*it.*, *Colli Albani*), and to the east by the Lepini and Ausoni Mountains (*it., Monti Lepini, Monti Ausoni*).

The ancient town at *Norba* (Google Earth DMS coordinates: 41°35′26″ N and 12°57′40″ E; Fig. 1) lies on a small plateau, bounded towards the north and east by the steep slopes of the Lepini Mountains, consisting of Neogene limestones (Peccerillo 2017). To the south and west, the plateau grades into gentle sloping hills made of volcanic tuffs, with volcanic glass fragments (Giordano et al. 2006; Giordano and The CARG Team 2010). North-west of *Norba* are the Alban Hills, with a Pleistocene quiescent volcano. The volcanic rocks in the Alban Hills comprise mainly pyroclastic material and lavas, but also Si-undersaturated leucite-bearing rocks, as well as a few melilite-bearing rocks. Main phenocrysts include clinopyroxene, leucite, and K-feldspar, as well as some melilite and garnet, and rare plagioclase (Giordano and The CARG Team 2010; Peccerillo 2017). During the Mid-Pleistocene, thick paleosoils developed on weathered pyroclastic material. They exhibit prominent translocation of reddish-brown clay and are accompanied by residual accumulation of Fe- and Mn-rich aggregates (Ugolini and Dahlgren 2002).

### Archaeological background

The Pontine Region was part of ancient *Latium* (present-day Lazio). Roman colonisation in this area began in the sixth century BCE. Two examples of colonised settlements are *Norba*, which was founded as a Latin colony in 492 BCE, and *Satricum* (present-day Borgo Faiti), which was strategically located along the Astura River, ca. 8 km from the Tyrrhenian Sea (Fig. 1). Excavations here have revealed a monumental temple dedicated to *Mater Matuta*, as well as three pottery kilns, which were positioned around the place of worship and were active between the sixth and fourth centuries BCE (Revello Lami 2017; Nijboer et al. 1995). Numerous depositions, comprising locally made anatomical votives and coarse ware, point to the enhanced experience of combined ritual and craft practices (Bouma 1996).

It was mainly from the fourth century BCE that existing towns and rural settlements expanded and new infrastructure was built in the Pontine region. For instance, *Norba* developed into a considerably sized town during the fourth and third centuries BCE, while the construction of the *Via Appia* favoured the development of various roadside settlements, such as *Forum Appii*, in the Inner plain (Fig. 1; Tol et al. 2021; De Haas 2017a, b). Archaeological surveys around *Forum Appii* have indicated that the site may have incorporated a harbour, as as well several pottery kilns where ceramic building material (e.g. tiles, cover tiles) and amphoras were manufactured during the second and first centuries BCE (Tol and Borgers 2016; Borgers et al. 2018). It is possible, however, that the construction of the *Via Appia* contributed to the abandonment of *Norba* after the town was destroyed during the Civil War in 82 BCE (De Haas 2011).

Archaeological surveys have also been carried out in the surroundings of *Norba*. These surveys have permitted to map a dense pattern of Roman Republican sites along the foothills of the Lepini Mountains, and more dispersed farmsteads and villas in the uplands, north of *Norba* (Van Leusen et al. 2003/2004, 2009/2010; De Haas 2011; De Haas et al. 2011/2012). The results further suggest that large villa estates developed in the third century BCE, which were involved in commercial activities related to agriculture (Attema and De Haas 2005; De Haas et al. 2011/2012). At one of these third century BCE villa domains, evidence for local pottery production has been found, including a kiln structure, a kiln spacer, pottery waste of black gloss fine ware, and coarse ware flagons and bowls (Van Leusen et al. 2003/2004, 2009/2010). The activity of the workshop has been dated to the second century BCE (Tol and De Haas 2013). Unfortunately, these objects have neither been collected nor preserved. Consequently, they could not be investigated in this research.

## Materials and methods

Materials. The Roman Republican ceramic material from the surveys around *Norba* covers the typical repertoire of rural settlement assemblages, comprising black gloss fine ware, amphora fragments, and coarse ware, such as jars (Tol 2017). Characteristic coarse ware jars incorporate two main types, henceforth referred to as olla type 2 and olla type 3a, following from the typo-chronology suggested by Olcese (2003). Both types of jars are common in Central Tyrrhenian Italy and occur in the assemblages from other Roman Republican sites in the Pontine region (e.g. Tol 2012, chapter 5 site 15106).

**Fig. 2:**
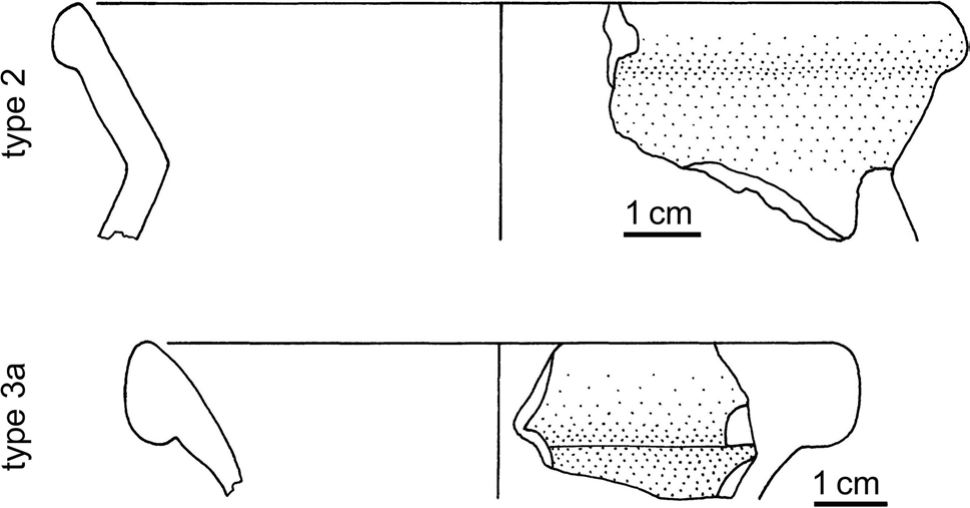
Type 2 and type 3a jars from *Norba* (redrawn from De Haas 2011, plate 30 no. 15 and plate 39 no. 19)

The shape of both types of jars is defined by an ovoid body and an almond-shaped rim, and the main difference between the two types is in the rim shape. More specifically, olla type 2 jars are defined by a high-collared rim (Fig. 2). The type is common in Southern Etruria and Rome from the sixth century BCE onwards. However, in the Pontine region and around *Norba*, the shape starts to appear in the fourth century BCE (Tol 2012; Borgers et al. 2017). In comparison, olla type 3a jars display a pronounced almond-shaped thickening below the lip (Fig. 2). These jars start to circulate in the study region from the late third or early second century BCE, and are considered to be the successor of the type 2 jars (Olcese 2003, type 3a, tav. VIII).

A total of 32 samples were selected from the diagnostic rim fragments, which have been collected during the surveys; they include 22 samples of type 2 jars, and 10 fragments of type 3a jars (Table 1).

**Table 1:** Macrogroups (MG) and predominant microscopic components of the 32 coarse ware jars from the archaeological surveys around *Norba*, including the optical characteristics of the matrix (Mx)

Sample no	Type	Site ID	MG	Mx	Sorting	Munsell values for HUE 5 YR		OM														
						Surface	Core	Cpx	Kfs	Bt	Qz	Lct	Ms	Chert	Pl	Volc	Amp	Fe-pellets	OI	Pumice	Silt	Petrogr
NO1	3a	1	4	≈Δ to ↓Δ	Moderate	Yellowish red 5/8		•	•	•	•				•	•		•	•	•		4
NO2	3a	1	3	Is	Moderate	Reddish yellow 7/8	Dark gray	•	•		•				•				•			3
NO3	3a	1	1	≈Δ	Moderate	Yellowish red 4/6		•	•	•	•	•	•	•		•	•	•	•	•		1
NO4	3a	1	4	≈Δ to ↓Δ	Moderate	Yellowish red 4/6		•	•	•	•					•		•	•	•		4
NO5	2	9	3	Is	Moderate	Reddish yellow 6/8	Very dark gray 3/1	•	•		•				•							3
NO6	3a	9	3	↓Δ	Moderate	Reddish yellow 6/8		•	•		•				•							3
NO7	2	11	1	↑Δ	Poor	Yellowish red 5/8	Very dark gray 3/1	•	•	•		•	•	•			•	•	•			1
NO8	2	11	1	↓Δ	Poor	Yellowish red 5/8	Very dark gray 3/1	•	•	•	•		•		•		•	•	•	•		1
NO9	2	11	2	≈Δ	Moderate	Light reddish-brown 6/4		•	•	•	•			•	•						•	2
NO10	2	11	1	↓Δ	Poor	Yellowish red 4/6	Gray 5/1	•	•	•	•	•	•	•			•	•	•			1
NO11	2	11	1	↓Δ	Poor	Yellowish red 5/8	Dark gray 4/1	•	•	•		•	•	•			•	•	•	•		1
NO12	2	11	1	↑Δ	Poor	Yellowish red 4/6	Very dark gray 3/1	•	•	•	•	•	•	•		•	•	•	•			1
NO13	2	11	1	↓Δ	Poor	Yellowish red 4/6	Very dark gray 3/1	•	•	•	•	•	•	•		•	•	•	•			1
NO14	2	11	2	↑Δ	Moderate	Reddish yellow 7/6	Gray 5/1	•	•	•	•			•	•						•	2
NO15	2	11	2	↓Δ	Moderate	Reddish yellow 7/6		•	•	•	•			•	•						•	2
NO16	2	11	1	↑Δ	Poor	Yellowish red 4/6	Gray 5/1	•	•	•	•		•		•		•	•	•			1
NO17	2	11	1	↓Δ	Poor	Yellowish red 4/6	Very dark gray 3/1	•	•	•	•	•	•	•			•	•	•			1
NO18	2	11	1	↑Δ	Poor	Yellowish red 4/6	Very dark gray 3/1	•	•	•		•	•	•			•	•	•	•		1
NO19	2	11	1	↓Δ	Poor	Yellowish red 4/6	Very dark gray 3/1	•	•	•	•		•				•	•	•	•		1
NO20	2	11	1	↓Δ	Poor	Very dark gray 3/1		•	•	•	•	•	•				•	•	•	•		1
NO21	2	11	1	≈Δ	Moderate	Yellowish red 4/6	Dark gray 4/1	•	•	•	•	•	•	•			•	•	•	•		1
NO22	2	11	1	Is	Poor	Yellowish red 4/6	Very dark gray 3/1	•	•	•	•	•	•	•	•		•	•	•			1
NO23	2	11	1	≈Δ	Poor	Yellowish red 4/6	Very dark gray 3/1	•	•	•	•	•	•	•	•		•	•	•			1
NO24	3a	16	3	Is	Moderate	Reddish yellow (7/8)	Very dark gray 3/1	•	•	•	•									•	•	3
NO25	3a	?	1	↑Δ	Poor	Yellowish red 4/6	Very dark gray 3/1	•	•	•		•	•	•			•	•	•	•		1
NO26	2	16	1	↓Δ	Poor	Very dark gray 3/1		•	•	•	•	•	•				•	•	•			1
NO27	3a	16	1	↓Δ	Poor	Yellowish red 4/6	Very dark gray 3/1	•	•	•	•	•	•				•	•	•			1
NO28	3a	25	1	≈Δ	Poor	Yellowish red 4/6 Very dark gray 3/1		•	•	•	•		•	•	•		•	•	•	•		1
NO29	2	26	1	Is	Poor			•	•	•	•		•				•	•	•	•		1
NO30	3a	25	1	Is	Poor	Reddish yellow (7/8)	Very dark gray 3/1	•	•	•	•	•	•				•	•	•	•		1
NO31	2	26	1	↑Δ	Moderate	Yellowish red 5/8		•	•	•	•	•	•	•			•	•	•			1
NO32	2	26	1	≈Δ	Moderate	Yellowish red 5/8		•	•	•			•	•			•	•	•			1

↑Δ high birefringence, ≈Δ moderate birefringence, ↓Δ low birefringence, *Is* isotropic. Sorting, surface, and core colour (Munsell 1994). *Cpx* Clinopyroxene, *Kfs* K-feldspar, *Qz* quartz, *Pl* Plagioclase, *Lct* Leucite, *Bt* Biotite, *Volc* volcanic rock, *Amp* Amphibole, *Ms* muscovite, *OI* opaque inclusions, *Silt* siltstone, *Petrogr* petrogroup

●Present; Mineral abbreviations acc. to Warr (2021)

### Methods

The first stage of analysis was conducted at the macroscopic scale, to gauge broad groups based on inclusion variability, sorting, and abundance. This was complemented by recording the colour and uniformity of colour on vessel surfaces and fresh breaks, using Munsell charts (1994), with the aim to gain basic information on firing conditions (Kreimeyer 1987; Nodari et al. 2004; Maritan et al. 2006; Rathossi and Pontikes 2010; Noghani and Emami 2014; Laita and Bauluz 2018).

In the second stage, fragments were studied in polarised light optical microscopy (OM), X-ray powder diffraction (XRD), and cold field emission scanning electron microscopy coupled with energy-dispersive X-ray spectrometry (CFE-SEM-EDX), to examine various aspects of the production technology of coarse ware, including raw materials, paste recipes, and firing processes. All 32 selected fragments were examined in OM. However, some samples were so small that XRD and CFE-SEM-EDX analysis could only be performed on 20 samples and 19 samples, respectively.

Standard petrographic thin sections were prepared from slices cut from each sample. They were studied with a Leica DM4500 P polarised light microscope (Department of Lithospheric Research, University of Vienna) and with an Axio Imager.A2m Zeiss polarised light microscope (Electron Microscopy Center at Babeş-Bolyai University Cluj-Napoca). The images were captured with a Zen 2011 Axio high-resolution digital video camera. The matrix and the inclusions of the ceramic thin sections were examined by OM (Table 1), in order to reconstruct specific aspects of the technology, including paste recipes and firing temperature (Quinn 2013, 2022). Inclusions smaller than 15–20 μm are taken to be naturally embedded within the clay (Maggetti 1979, 1982; Ionescu et al. 2011a). The size, shape, quantity, uniformity, and sorting of the coarse-sized grains were useful criteria to detect specific paste recipes, such as the addition of temper.

XRD was performed on 20 samples (NO1, NO3, NO4, NO6, NO8–NO10, NO12–NO17, NO19, NO21–NO24, NO28, NO31), to establish mineralogical compositions and to identify changes in the ceramic body, which might have occurred during the firing process (Ionescu et al. 2011b; Gál et al. 2018; Borgers et al. 2022). A few grams of each sample were hand-milled in an agate mortar and analysed with a Bruker D8 Advance diffractometer with Bragg-Brentano geometry, working with Cu-radiation at 40 kV and 40 mA, at the Department of Geology, Babeş-Bolyai University, Cluj-Napoca. The scanning interval was between 5 and 64° 2θ, with a 0.02° 2θ step. Corundum NIST SRM1976a was used as standard. The minerals were identified with Bruker’s Diffrac.Eva 2.1 software and ICDD PDF 2016 database.

CFE-SEM-EDX was used to examine the microstructure of the matrix and firing products, with the aim to estimate the firing process of ancient pottery (Maniatis and Tite 1981; Tite et al. 1982; Cultrone et al. 2004; Maritan et al. 2005; Gál et al. 2018; Borgers et al. 2020, 2022). For this purpose, 19 samples (NO1, NO3, NO4, NO6, NO8, NO10, NO12–NO17, NO19, NO21–NO24, NO28, NO31) were examined with a Hitachi 8230 microscope working at 30 kV acceleration voltage, 50 s live time and with a < 10 nm electron beam, allowing a very high resolution of the backscattered and secondary electron images. The surface of freshly fractured samples was coated with Au for image capture, but the surface of the samples was not coated for chemical analysis. Data for major oxides, including SiO_2_, TiO_2_, Al_2_O_3_, Fe_2_O_3_ as FeO_TOT_, MgO, CaO, K_2_O, Na_2_O, P_2_O_5_ and MnO were obtained by EDX. The detection limit was ~ 0.1 wt.% for all oxides.

In the third stage, the compositional groups of the fourth–first centuries BCE coarse ware defined in this study were compared with published work on coarse ware from various sites in Rome and its suburbs (Schuring 1986, 1987; Olcese 2003; Thierrin-Michael 2003; Borgers and Fischetti 2023), from Ostia (Capelli 2016), and from Rome’s rural hinterland (Borgers et al. 2017, 2018), to understand whether the surroundings of *Norba* were integrated into the same regional production and trade networks.

In the fourth and final stage, the Roman Republican coarse ware from *Norba* was compared with geological field samples from the Pontine region, which have been collected and investigated in a previous research project (Borgers et al. 2018), in order to identify possible raw materials used for the production of coarse ware.

## Results

### Macroscopic analysis

Based on the type, size, abundance, and sorting of coarse inclusions, as well as the colour visible on the surface and in the fresh break of the sherds, four macrogroups were defined within the assemblage analysed (Table 1).

**Fig. 3:**
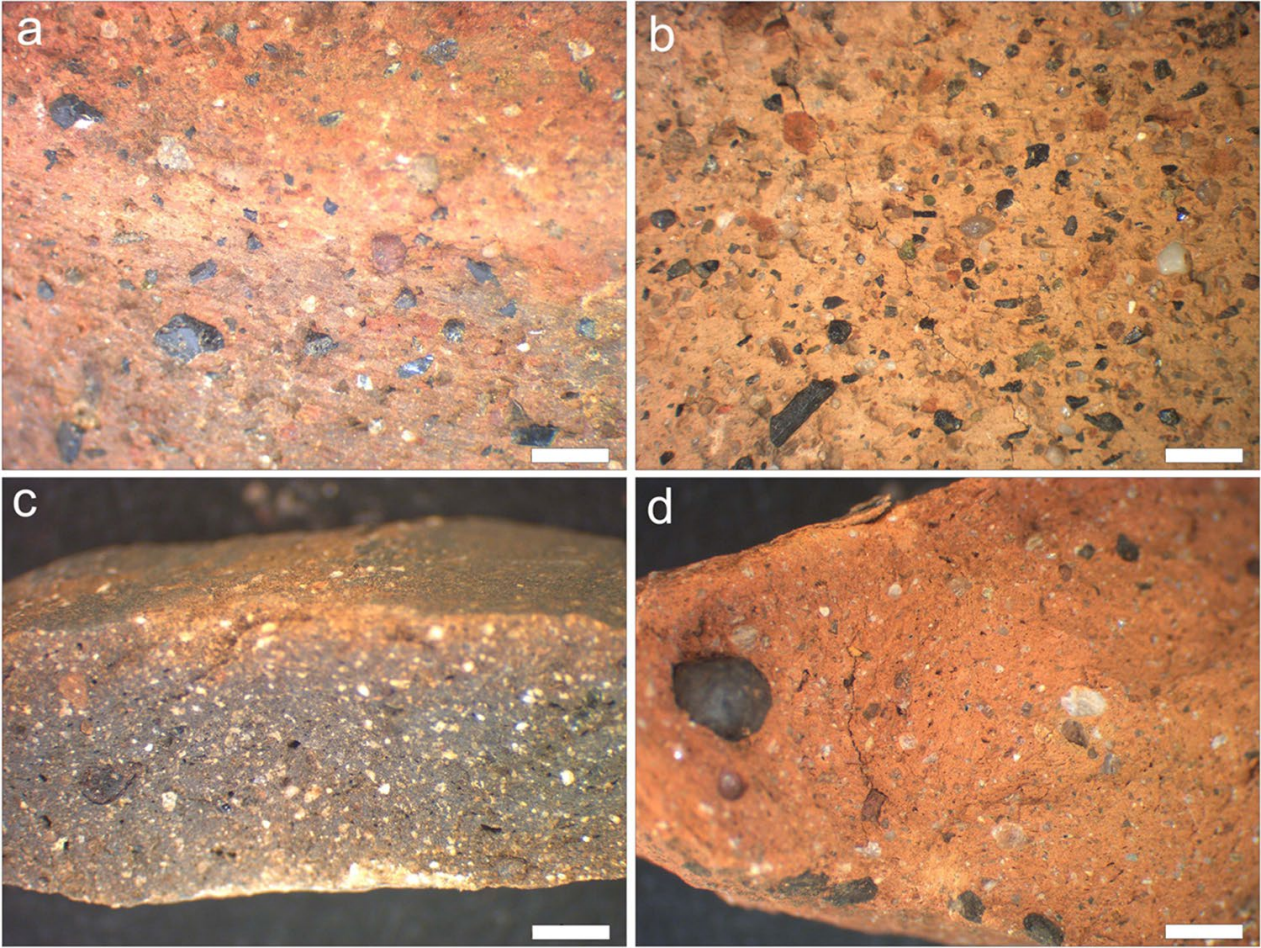
Images of the four macrogroups identified in the coarse ware assemblage from *Norba*, based on the type, sorting, and abundance of coarse inclusions: **a** Macrogroup 1: coarse pyroxene and K-feldspar in sample NO8. **b** Macrogroup 2: coarse quartz, K-feldspar and pyroxene in sample NO15. **c** Macrogroup 3: moderately sorted K-feldspar and quartz in sample NO5. **d** Macrogroup 4: poorly sorted coarse K-feldspar, rounded opaque (Mn or Fe) aggregates, and pyroxene in sample NO1. Scale bar = 2 mm for all images

*Macrogroup 1* contains 23 samples, and displays predominantly coarse pyroxene and K-feldspar, varying in size between 500 µm and 1 mm. Most of the samples in this group (*N* = 16) display a reddish yellow or yellowish red colour on the surface and a gray to very dark gray colour in the core, suggesting that the firing process was not long enough for oxygen to fully penetrate in the clay body (Fig. 3a). Four samples have a reddish colour, resulting from consistently oxidising firing conditions, while three samples are gray, indicating a firing process in reducing atmosphere with a higher proportion of carbon (Laita and Bauluz 2018).

*Macrogroup 2* comprises three samples that are defined by coarse rounded quartz and K-feldspar, with sporadic biotite and pyroxene, ranging between 300 and 500 µm. The coarse inclusions comprise ca. 45% of the clay body, indicating that the potter may have deliberately added them as temper. Two samples are reddish yellow to light reddish brown (Fig. 3b), following from firing in a well-controlled oxidising atmosphere (Kreimeyer 1987; Rathossi and Pontikes 2010), while the third fragment displays a sandwich structure with a reddish yellow surface and gray core colour, indicating, among other variables, that the oxidising firing process was comparatively short (Nodari et al. 2004; Maritan et al. 2006; Noghani and Emami 2014).

The four samples in *macrogroup 3* are defined by moderately sorted K-feldpar and quartz (250-300 µm), with pyroxene. Three samples display a sandwich structure, suggestive of an incomplete firing in oxidising atmosphere (Fig. 3c; Nodari et al. 2004; Maritan et al. 2006; Noghani and Emami 2014), while the fourth sample is reddish, resulting from firing in consistently oxidising conditions (Rathossi and Pontikes 2010).

The two samples of *macrogroup 4* display very few coarse, poorly-sorted K-feldspar and rounded opaque inclusions (< 2 mm; Fe- or Mn-rich aggregates), as well as scarce coarse pyroxene (Fig. 3d). The firing conditions were consistently oxidising, given that the samples have a reddish colour both on the surface and in the core (Kreimeyer 1987; Rathossi and Pontikes 2010).

### Optical microscopy

The ceramic samples have been classified into four petrogroups, based on the observation performed under the polarized light microscope. All four groups are associated with the macrogroups (Table 1). The description of the main characteristics of the groups follows below, and the mineral compositions are listed in Table 1.

All 32 ceramic thin sections comprise fine-sized (15–20 µm) grains of quartz, K-feldspar, biotite, and occasional plagioclase, which are embedded within the matrix. In the ceramic mass, there are also inclusions larger than 100 µm, generally poorly (e.g. petrogroups 1, 2, and 4) to moderately sorted (e.g. petrogroup 3). Most samples comprise coarse-grained inclusions in the range of 150–500 µm. Occasionally larger inclusions (1 mm) are present. Only few samples are fine (large inclusions, ca. 300 µm, are very rare).

**Fig. 4:**
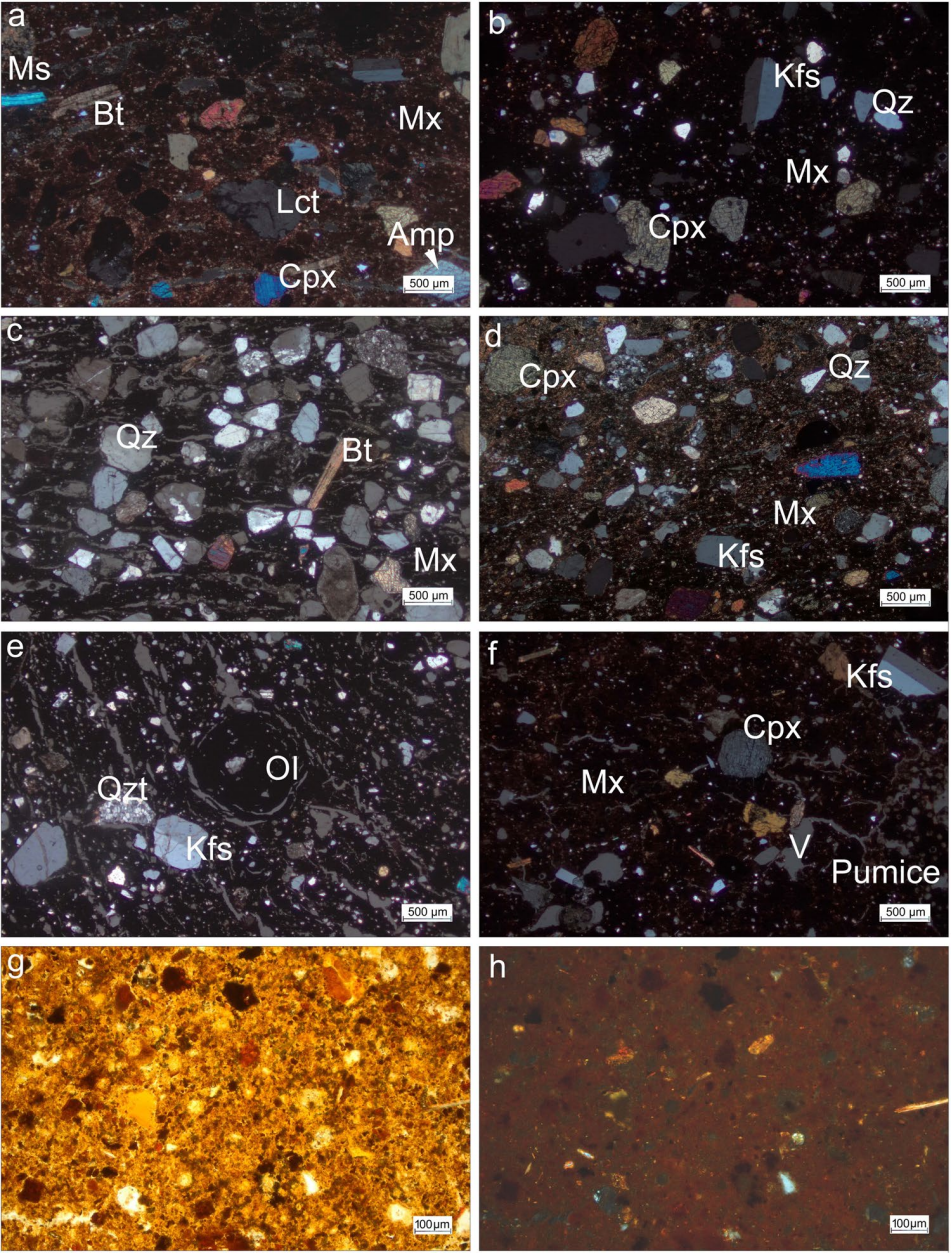
Optical micrographs with crossed polarizers of the four petrogroups and clay sample: **a**, **b** Petrogroup 1: clinopyroxene (Cpx), K-feldspar (Kfs), amphibole (Amp), leucite (Lct), muscovite (Ms), and biotite (Bt) in NO12 and NO19 respectively. **c** Petrogroup 2: quartz (Qz), biotite, and chert in NO15.** d** Pottery waste from *Satricum*: quartz, K-feldspar, and clinopyroxene. **e** Petrogroup 3: K-feldspar, quartz, opaque inclusion (OI) with shrinkage rim, and quartzite (Qzt) in NO2. **f** Petrogroup 4: K-feldspar, clinopyroxene, and pumice in NO4. **g**, **h** Clay sample of paleosoil on weathered tuff in PPL and XP, respectively. For images **a** to **f**, the polarizers were crossed at ≠ 90 °for a better distinction between matrix (Mx; very dark hue) and voids (V; light grey; elongated and irregular shape)

*Petrogroup 1* (Fig. 4a, b) is the largest group in the studied assemblage, comprising 23 samples. They are characterized by large ubiquitous sub-angular clinopyroxene (500 µm–1 mm), angular K-feldspar (500 µm), and rounded Fe-rich clay pellets, varying in size between 150 and 300 µm. Large (up to 1 mm in size), more or less spherical, opaque (Mn- or Fe-rich) inclusions with an internal concentrical structure, are characteristic. Coarse leucite (500 µm), biotite (200–500 µm), quartz, muscovite (200–300 µm), and amphibole (< 500 µm), as well as very rare plagioclase have also been identified. Very few rock inclusions are present, including chert, pumice (e.g. NO3), and other volcanic rock fragments (NO12, NO13, NO31). All the coarse inclusions in the sherds assigned to petrogroup 1 are sub-angular to rounded in shape and seem to have been added as temper. Most samples in this group are heavily tempered (ca. 35% inclusions *versus* ca. 65% matrix), while three samples (NO3, NO21, NO31) comprise comparatively little temper (ca. 20%). About half of the samples of petrogroup 1 display low birefringence (Table 1), suggesting a relatively high firing temperature. Five samples are characterized by moderate birefringence, indicating a medium firing temperature, while the six remaining samples show high birefringence, which is compatible with a low firing temperature. The samples from petrogroup 1 bear mineralogical similarities to the production waste from Tivoli (Thierrin-Michael 2003: 58).

The three samples from *petrogroup 2* (NO9, NO14, NO15) are defined by coarse rounded quartz (300–500 µm), and sub-angular K-feldspar (300–500 µm). Subangular clinopyroxene (250–500 µm), biotite (500 µm), subangular siltstone (300–500 µm), and subangular iron-stained chert (300–600 µm) are less frequent (Fig. 4c). The coarse-grained inclusions, consisting of a mixture of sedimentary and subangular volcanic grains, comprise ca. 45% of the ceramic body, suggesting that they have been deliberately added. The matrix of two samples (NO9, NO15) shows moderate to low birefringence, while the third sample (NO14) displays high birefringence. This indicates that the firing temperature of the samples in this group varies from medium to low, respectively. The type, size, and frequency of coarse inclusions present in petrogroup 2 samples are similar to the production waste from *Satricum* (Fig. 4d; Attema et al. 2003).

*Petrogroup 3* comprises four samples (NO2, NO5, NO6, NO24), and is characterised by moderately sorted K-feldspar and quartz, ranging between 250 and 300 µm (rarely 500 µm). Rare clinopyroxene and plagioclase have been identified, as well as scarce subangular quartzite (300 µm) (Fig. 4e). These coarse grains comprise about 20–25% of the ceramic body, suggesting that they are added tempering material. Rare spherical opaque (Mn- or Fe-rich) inclusions (500 µm), similar to those identified in petrogroup 1, were also found. The isotropic matrix of NO2, NO5, and NO24 and the low birefringence of NO6 indicate a high firing temperature for all four samples. The mineralogical composition of the samples from petrogroup 3 is similar to the local coarse ware from Vasanello, for which it has been suggested that the coarse-sized K-feldspar and quartz, as well as rare clinopyroxene and plagioclase, have been intentionally added by the potter (Peña 1992: 114).

The two samples in *petrogroup 4* (NO1, NO4) are defined by numerous Fe- or Mn-rich aggregates, and pumice fragments (> 500 µm) in the matrix (Fig. 4f). Very rare coarse angular K-feldspar (200–500 µm), subangular clinopyroxene (300–500 µm), and volcanic rocks, most likely volcanic glass (500 µm–1 mm), have also been found. Both samples are defined by a moderate to low birefringence, compatible with a medium to high firing temperature. The coarse inclusions comprise about 15% of the ceramic mass, indicating that they are naturally embedded within the clay. The conspicuous Fe/Mn-rich aggregates and pumice in the base clay bear broad similarities to the ceramic building material produced at the site of *Forum Appii* (Borgers et al. 2018), and to the paleosoil clays that develop on weathered tuffs (Ugolini and Dahlgren 2002; Fig. 4g, h).

### X-ray diffraction

**Fig. 5:**
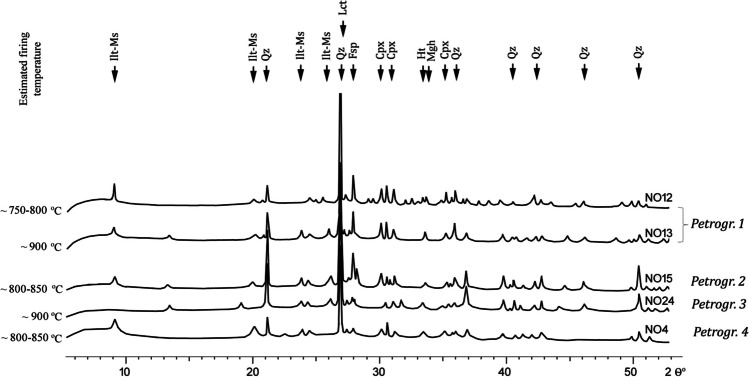
Representative diffractogram of each petrogroup with estimated firing temperatures. Illite-muscovite (Ilt-Ms), quartz (Qz), leucite (Lct), feldspar (Fsp, undifferentiated), clinopyroxene (Cpx, undifferentiated), hematite (Ht), and maghemite (Mgh)

The diffractograms obtained for the 20 samples analysed reveal the prevalence of quartz, feldspar, and clinopyroxene (Fig. 5)—minerals, which were also identified as coarse inclusions by OM (Table 1; Fig. 4a–f). In addition, the diffraction peaks (3.27 Å d-spacing) indicate the presence of leucite in the samples of petrogroup 1 only, which is in agreement with the evidence seen by OM (Fig. 4a, b). Further to this, the clinopyroxene peaks (2.99 Å d-spacing) are evident in all samples, except for petrogroup 3, and can be identified as augite and diopside. The presence of neoformed clinopyroxene, referred to as “ceramic pyroxene” in the literature (Dondi et al. 1998; Gliozzo 2020), is possible but difficult to confirm based on XRD results alone. Small peaks with d-spacing 2.7 Å and 2.5 Å might be hematite and maghemite respectively.

Illite and muscovite have partially overlapping peaks (with 10, 5, 4.5, and 2.6 Å d-spacing), due to their similar structural unit (Gliozzo 2020). Consequently, the clays used to produce the coarse ware jars in this study are henceforth referred to as ‘illite-muscovite’ (Gál et al. 2018). Most peaks (in particular, 10 Å d-spacing) are either low or missing, while few are comparatively high (Fig. 5). This corresponds to the birefringence/isotropy of the clayey matrix observed by OM. More specifically, a low temperature of 750–800 °C can be associated with a high birefringence of the matrix, a medium temperature of 800–850 °C with a moderate birefringence, and a high temperature of ~ 900 °C corresponds to a low birefringence (Cultrone et al. 2001, 2004; Gliozzo 2020; Montana 2020; Quinn 2013, 2022).

### Cold field emission scanning electron microscopy with energy-dispersive X-ray spectrometry

CFE-SEM-EDX was used to examine the microstructure and composition of the clayey matrix, the firing products, and the nature of the various inclusions.

#### Microstructure of the matrix

**Fig. 6:**
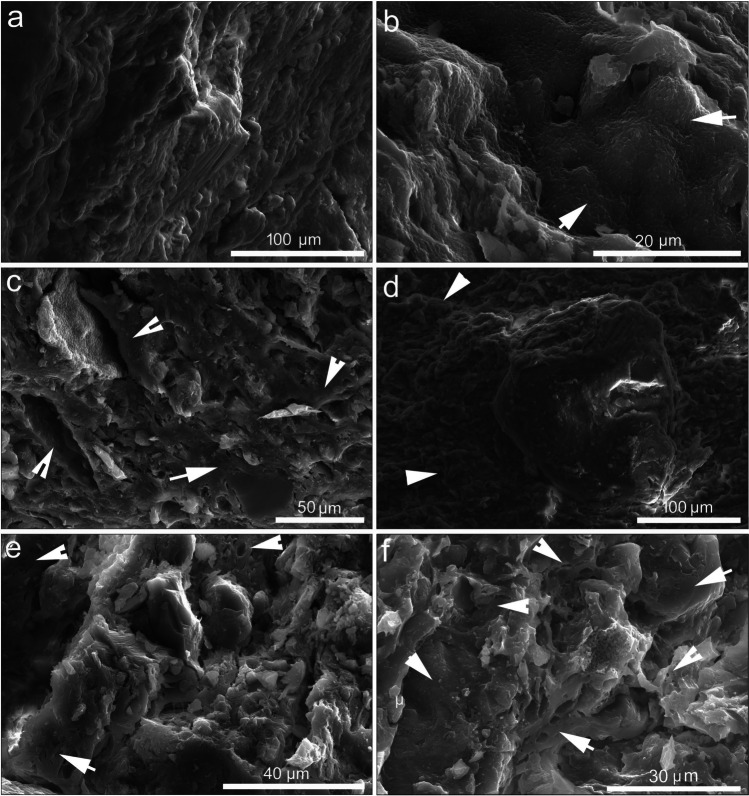
Secondary electron images of the matrix. Petrogroup 1: **a** Sintered matrix with sheetlike phyllosilicates in NO31. **b** Vitrification in NO3. **c**, **d** Advanced vitrification visible in deformed phyllosilicates in NO13 and NO19; Petrogroup 2: **e** Vitrification visible through smoothed margins of phyllosilicates in NO9; Petrogroup 4: **f** Vitrification in NO4. The arrows point to vitrified areas

Following research by Maniatis and Tite (1981), Cultrone et al. (2004), Maritan et al. (2005), Gál et al. (2018), Borgers et al. (2020, 2022) a.o. on microstructural changes of ceramic bodies, significant differences were identified among the coarse ware jars studied. The matrix of the samples NO12, NO16, and NO31, assigned to petrogroup 1, shows sintering in the form of very thin films (~ 5 µm) of interconnecting phyllosilicates, which have maintained their sheetlike structure (Fig. 6a). The sintering is indicative of a low firing temperature (750–800 °C) (Maniatis and Tite 1981; Tite et al. 1982). Other samples from petrogroup 1 (NO3-Fig. 6b, NO8, NO10, NO22, NO23, and NO28) show vitrification, revealed by comparatively larger areas (20 µm) of melted particles, indicating a firing temperature of 800–850 °C. Samples NO13 (Fig. 6c), NO17, and NO19 (Fig. 6d) display advanced vitrification, with large glassy areas and deformed and clumped phyllosilicates (50 µm), suggesting a firing temperature of at least 900 °C.

The samples from petrogroup 2 display evidence for sintering (NO14) and vitrification (NO15, NO9; Fig. 6e), suggestive of a firing temperature at 750–850 °C and 800–850 °C, respectively. Advanced vitrification is characteristic of petrogroup 3 (NO6 and NO24), which is confirmed by OM analysis by the shrinkage rims around opaque inclusions (Fig. 4e) that have formed due to their contraction during cooling. The two samples from petrogroup 4 display vitrification (NO4; Fig. 6f) and advanced vitrification, indicating a firing temperature of 800–850 °C and 900 °C, respectively.

**Fig. 7:**
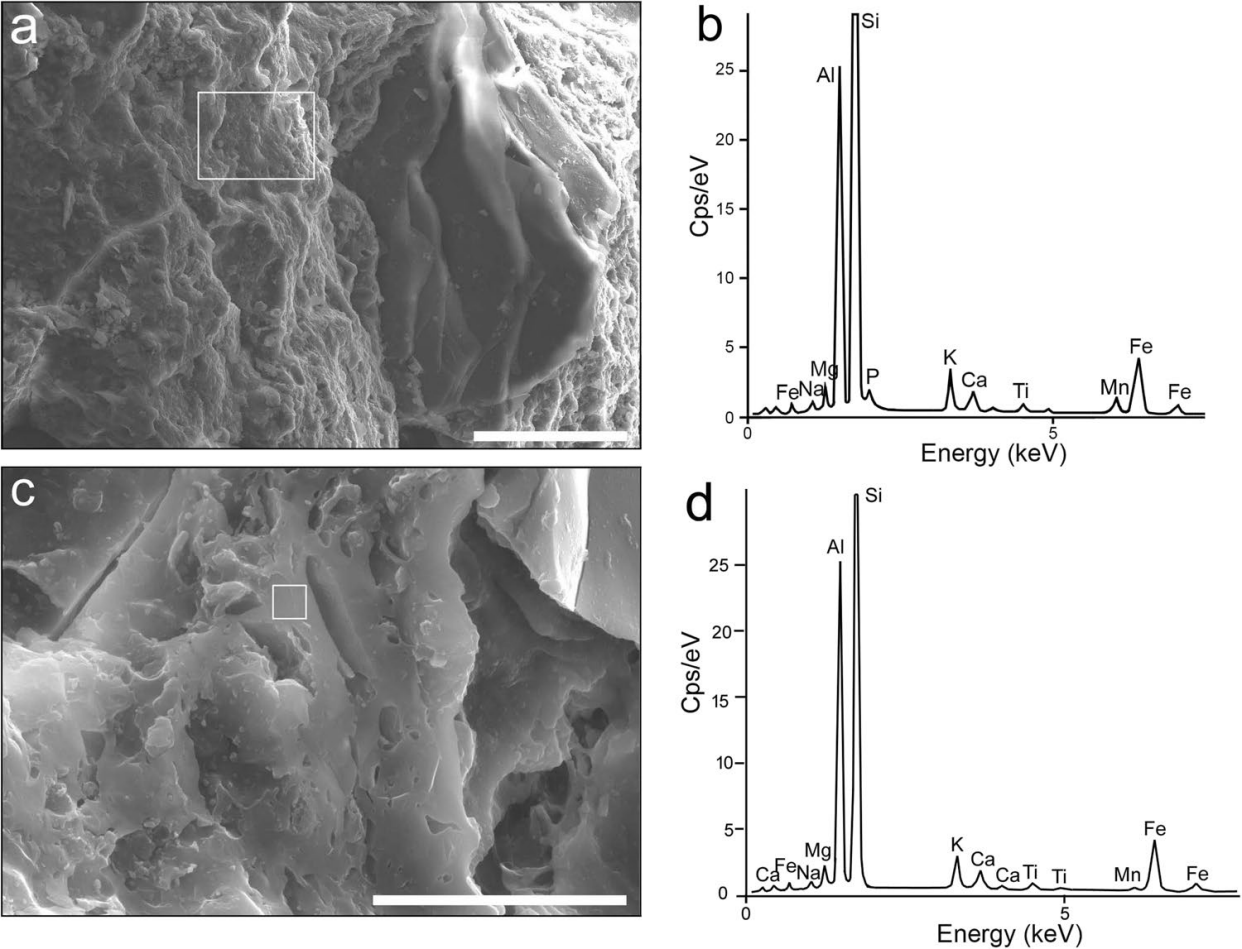
Secondary electron images and EDX spectra of clayey matrix (**a**, **b**) in NO3 and glass (**c**, **d**) in NO1. Scale bar is 100 μm for **a**, and 5 μm for **c**. The white rectangles mark the measurement areas

Table 2 illustrates compositional similarities, as well as significant differences, in the clayey matrix of the four petrogroups. Generally, for all petrogroups, the matrix is siliceous (> 50 wt.% SiO_2_), with relatively similar K, high Al and Fe, and low Ca (i.e. below 5 wt% CaO (Maniatis et al. 1981; Gliozzo 2020) (Fig. 7a, b). Petrogroup 1 displays high Al, Fe, and Ti, while petrogroup 2 is defined by the highest Fe and Ti and lowest Al, compared with the other groups. Petrogroup 3 displays the highest Si content, whereas petrogroup 4 shows the highest Al, paired with relatively high Ca.

**Table 2:** The variation limits for the main chemical compounds (expressed as oxides in wt.%) of the matrix, as determined by EDX

	SiO_2_	Al_2_O_3_	Fe_2_O_3_	K_2_O	CaO	TiO_2_
Petrogr. 1	48.1–66.6 (*54.8)	17.1–39.8 (*28.0)	4.2–23.1 (*10.3)	0.7–3.2 (*2.2)	0.5–2.6 (*1.6)	1.1–2.8 (*1.6)
Petrogr. 2	44.2–61.5 (*54.5)	19.7–30.0 (*26.5)	3.6–15.2 (*10.5)	2.0–5.3 (*3.4)	2.0–5.3 (*3.3)	1.0–2.6 (*1.8)
Petrogr. 3	46.6–71.2 (*58.6)	21.4–38.0 (*29.2)	5.5–13.4 (*8.0)	1.9–4.2 (*2.8)	0.7–1.1 (*1.0)	0.5–2.6 (*1.1)
Petrogr. 4	44.6–70.6 (*53.3)	22.1–38.3 (*31.8)	2.1–12.6 (*8.1)	1.7–2.2 (*2.2)	0.6–5.1 (*3.0)	0.4–1.2 (*1.0)

*Average composition

#### Firing products

**Fig. 8:**
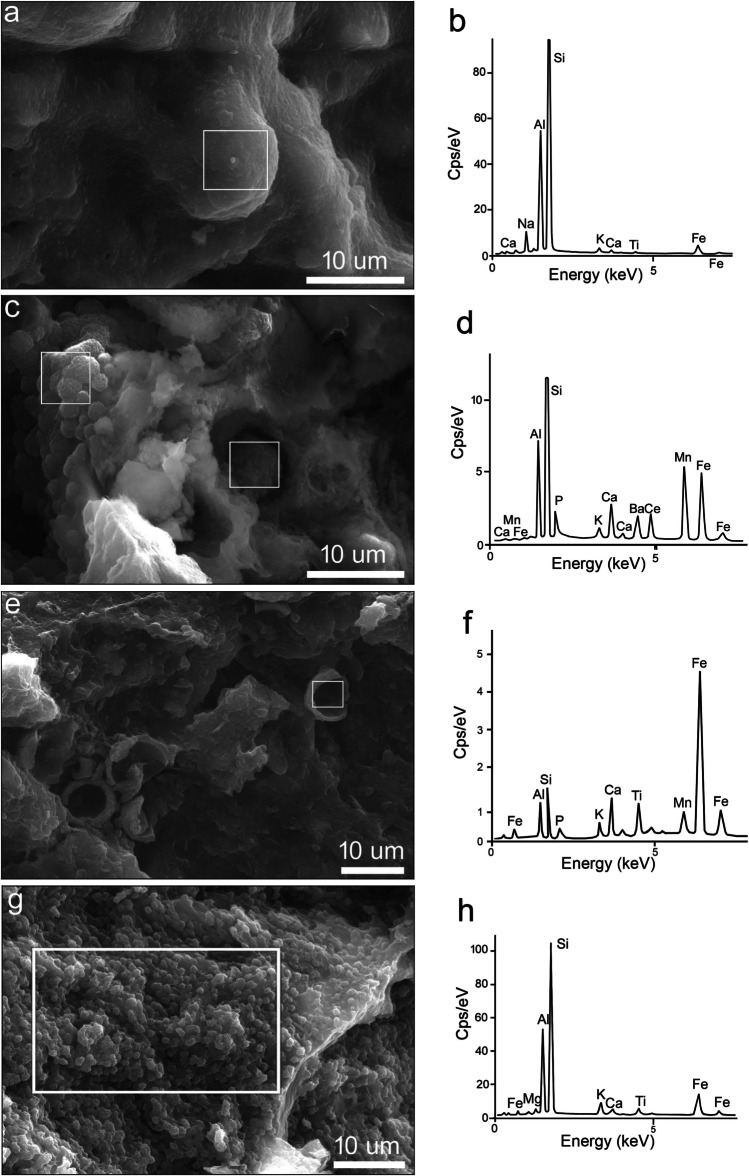
Textural and mineralogical changes during firing: **a**, **b** Glass resulting from the transformation of plagioclase, with corresponding EDX spectrum in NO13; **c**, **d** Mn- and Fe-rich aluminosilicate microspheres with EDX spectrum in NO19; both measured areas have the same spectrum; **e**, **f** Hemispheric formations with EDX spectrum in NO4; **g**, **h** Clusters of isometric Fe-rich aluminosilicate crystals (i.e. ‘ceramic pyroxene’) with EDX spectrum in NO13. The white rectangles mark the measurement areas

The *vitreous material* is an aluminosilicate glass with Fe and K, as well as small amounts of Ca, Ti, Mg, Mn, and Na (Fig. 7c, d)—its composition is similar to that of the matrix (Fig. 7a, 7b). The Si–Al–Na-rich glass, shown in Fig. 8a, b, might result from the transformation of a plagioclase feldspar inclusion.

Figure 8c shows microspheres (1.5 μm in diameter) with wrinkled surface, forming clusters, in NO19. A similar but larger (5 μm) microsphere occurs isolated nearby. They have an aluminosilicate composition with ~ 30 wt% SiO_2_ and 25 wt% Al_2_O_3_ (Fig. 8d), high Mn and Fe (~ 17 wt% MnO and ~ 12 wt% FeO_TOT_), as well as some P, Ba, and Ce. These microspheres might have formed from the reaction of the clayey matrix with Fe- and Mn-rich aggregates during firing. Fe-rich clay deposits, with Fe- and Mn-rich nodules, occur in the colluvial clay deposits in the Pontine region (Sevink et al. 1984; Borgers et al. 2018), and the paleosoils in the Alban Hills (Ugolini and Dahlgren 2002).

In samples NO1 and NO4 (Fig. 8e), hemispheric shapes were observed, measuring approximately 5–6 μm in diameter. Their composition is dominated by iron (50.8 wt.% FeO_TOT_), followed by aluminium (13.2 wt.% Al_2_O_3_) and silica (11.9 wt.% SiO_2_). Titanium (7.7 wt.% TiO_2_), manganese (6.5 wt.% MnO), and calcium (6 wt.% CaO) are also high (Fig. 8f). These hemispheres might have formed from the transformation of the Fe-rich pellets containing Ti present in the base clay.

Apart from vitrified areas, partly-melted material was also observed in sample NO13. It forms thin layers (of a few μm) around voids, and its surface is covered by clusters of small isometric grains, about 1 µm in size each (Fig. 8g). The grains consist of 52.0–57.5 wt.% SiO_2_, 26.9–28.3 wt.% Al_2_O_3_, 8.4–12.1 wt.% FeO_TOT_, 2.3–2.6 wt.% K_2_O, 1.3–1.6 wt.% MgO, 1.1–1.9 wt.% TiO_2_, 0.7–1.6 wt.% Na_2_O, and 0.8–1.1 wt.% CaO (Fig. 8h). Probably, these isometric grains are incipient crystals of ‘ceramic pyroxene’ formed during firing from the partly-melted material. Based on the low Ca content and the high Fe and Al content, it is likely that they are augite-type pyroxenes (Dondi et al. 1998; Gál et al. 2018; Pérez-Monserrat et al. 2022).

#### Inclusions

**Fig. 9:**
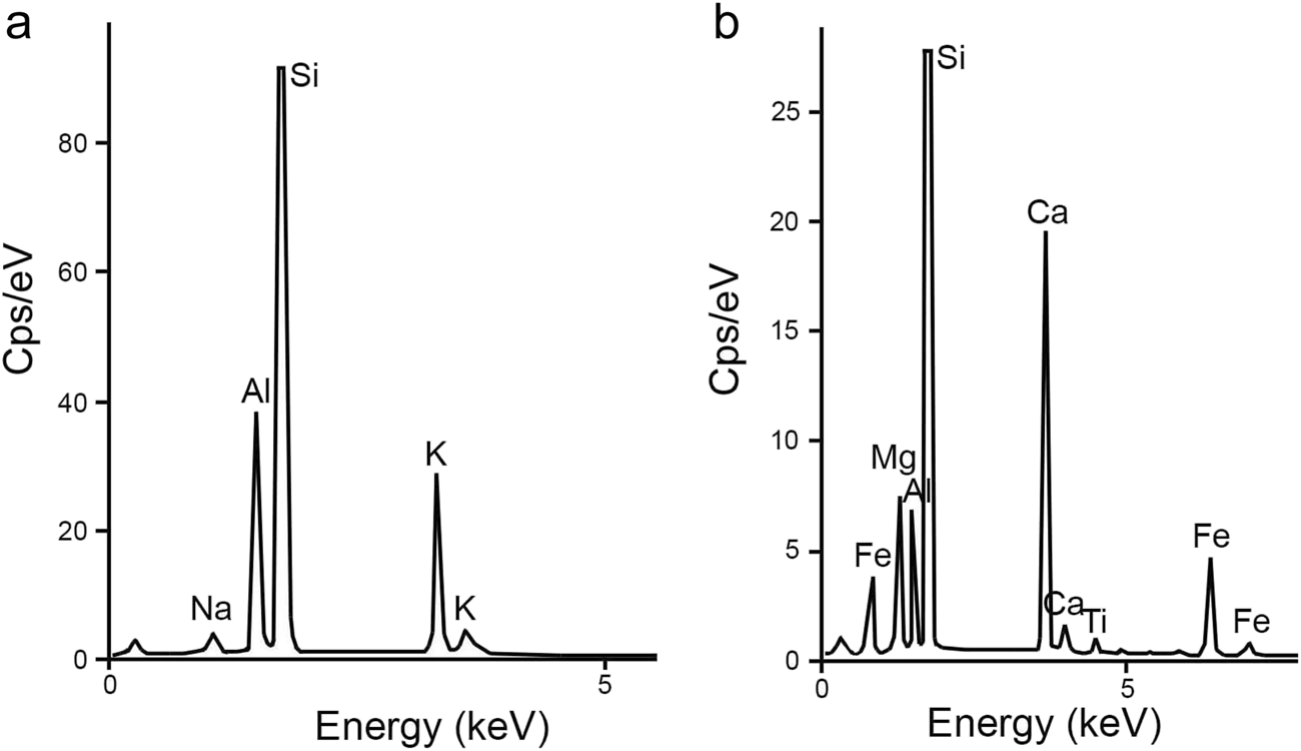
EDX spectra of inclusions in the ceramics: **a** K-feldspar in NO1. **b** Clinopyroxene in NO10

Besides matrix and firing products, EDX helped to identify various primary inclusions, such as K-feldspar (Fig. 9a), alkali feldspar (with significant Na content), clinopyroxene (Fig. 9b), plagioclase (albite), leucite, biotite, garnet, as well as Fe- and Mn-rich aggregates. Melilite was found in samples from petrogroup 1 only. All these minerals are specific to the volcanic area of the Alban Hills (Giordano and The CARG Team 2010; Peccerillo 2017). What is more, clinopyroxene was found in all four petrogroups, albeit scarce in petrogroup 3. Its variable mineralogy, ranging from diopside to augite and hedenbergite, is in agreement with published data on clinopyroxene from the Alban Hills (Aurisicchio et al. 1988; Boari et al. 2009; Conticelli et al. 2010; Giordano and The CARG Team 2010; Peccerillo 2017).

## Discussion

Based on the results of the multi-analytical study, the production technology of Roman Republican coarse ware from surveys around *Norba*, Southern Lazio, will be discussed. This will be followed by a preliminary reconstruction of possible trade of the coarse ware studied, based on a comparative study with previous data.

### Technology

All the coarse ware jars studied were produced with Fe-rich, Ca-poor, and illite-muscovite clays. Most jars display a sandwich structure, with a reddish colour on the surface and a gray colour in the core, suggesting that the firing process was not long enough for oxygen to fully penetrate the clay body. Very few samples have a homogeneous reddish colour, indicating that the firing conditions were consistently oxidising, or display a gray colour, suggestive of a firing process in reducing conditions. The most important differences between the coarse ware samples analysed are the paste recipes, i.e. the presence of coarse-grained inclusions, which were added by potters:

- The jars from petrogroup 1 are defined by clinopyroxene, K-feldspar, leucite, as well as some melilite—these minerals are common in the clayey deposits in the Alban Hills, and their presence suggests that the coarse ware from this group was produced in this area. This group bears compositional similarities to the production waste from Tivoli (Thierrin-Michael 2003: 58), but it should be noted that this workshop was active during the second–first centuries BCE, and produced type 3a coarse ware jars only (Olcese 2003: 15). If the coarse ware jars in this study, comprising both types 2 and 3a jars, were indeed produced at Tivoli, then it is reasonable to assume that the workshop began its activities earlier than is now known from excavations.
- The jars assigned to petrogroup 2 are tempered with rounded quartz and K-feldspar, sharing broad mineralogical similarities with the pottery waste from *Satricum*, where workshops where active until the fourth century BCE (Nijboer et al. 1995; Attema et al. 2003). This hypothesis is further supported by the fact that the coarse ware from petrogroup 2 comprises exclusively type 2 jars.
- The samples from petrogroup 3 display moderately sorted K-feldspar and quartz, as well as rare clinopyroxene and plagioclase. The composition of this group is similar to local coarse ware from the site at Vasanello (Peña 1992). Coarse ware with this composition has been grouped in the overarching ‘Rome and Tiber Valley Fabric’, on the assumption that pottery from other workshops along the Tiber River might have a similar composition (Olcese 2003).
- The two type 3a jars in petrogroup 4 are defined by pumice and Fe- or Mn-rich aggregates—i.e., these inclusions typically occur in paleosoils that developed on weathered pyroclastic material in the Alban Hills, as well as in colluvial clays located in the Inner plain of the Pontine region. With this in mind, the coarse ware may have been produced in one of two workshops active during the second–first centuries BCE: the first workshop was located near *Norba*. As mentioned, however, pottery waste from this workshop has not been preserved, and to the authors’ knowledge, it did not comprise any coarse ware jars. The second possible production centre is *Forum Appii*, located in the Inner plain of the Pontine region. Potters here used Holocene colluvial clay with pumice and Fe/Mn-rich aggregates for the manufacture of tiles and cover tiles (Borgers et al. 2018). If petrogroup 4 is indeed a local fabric from *Forum Appii*, then potters from this workshop also produced coarse ware jars.

### Distribution

The composition of the coarse ware jars in this study has been compared with previous data, to gain insight into trade networks, and changes therein between the fourth–first centuries BCE. More specifically, of the 22 samples of olla type 2 jars, dated to the Middle Republican era, 18 belong to petrogroup 1, and one fragment belongs to petrogroup 3. Following this, most jars were regional products, which were manufactured in the Alban Hills or in the Tiber Valley region, respectively. Both compositions were traded widely, given that:

- Coarse ware jars from petrogroup 1 were found on sites in Ostia (Capelli 2016: 196–198), and in Rome’s suburbs (Borgers and Fischetti 2023);
- Coarse ware jars from petrogroup 3 were found on various sites in Rome (Thierrin-Michael 2003; Schuring 1986, 1987), and its suburbs (Borgers and Fischetti 2023). Jars with this composition have also been found in Northern Italy (Peña 1992; Olcese 1990)

The three remaining type 2 jars belong to petrogroup 2, which seems to have originated in *Satricum*. Jars of this composition were distributed within the Pontine region, as they have been found on several sites in the Inner plain (Borgers et al. 2017).

The results in this study further point to changes in regional/local distribution networks during the Late Republican period (second-first centuries BCE). More specifically, of the 10 olla type 3a jars studied, five were imported from the Alban Hills, and three from the Rome and Tiber Valley. Hence, the two supra-regional trade networks that were in place during the Middle Republican era continued to exist during the Late Republican period. Differences in trade networks seem to have taken place at the regional level, however. This is illustrated on the one hand by the end of the production activity at *Satricum*, and on the other by the start of a new production, as reflected in petrogroup 4. Jars with this composition (*N* = 2), defined by conspicuous pumice and Fe/Mn-rich aggregates, seem to have been produced within the region – possibly at *Forum Appii* in the Inner plain of the Pontine region, or near *Norba*. Coarse ware with this composition has, to the authors’ knowledge, not been identified at other sites. This might suggest that they had a more restricted distribution pattern, but more research is needed to confirm this.

## Conclusions

Building upon previous data for coarse ware from Southern Lazio, Italy, this study examined 32 coarse ware jars from the surroundings of ancient *Norba* in the Lepini Mountains, dated between the Middle and Late Republican eras (fourth–first centuries BCE). A multi-analytical approach, combining macroscopic observation with OM, XRD, and SEM, was adopted to reconstruct aspects of the production technology of the coarse ware and to determine whether this area of Southern Lazio was integrated into similar trade networks as Rome and its rural hinterland.

Some interesting points emerge from this study. First, the results indicate that the coarse ware jars from *Norba* were manufactured with Fe-rich, Ca-poor, and illite-muscovite clay deposits and fired in incomplete oxidising conditions between 750 and 900 °C. These findings are in agreement with previous research conducted on coarse ware from Southern Lazio. From this is tentatively inferred that potters in the area shared knowledge of clay sourcing (e.g. Fe-rich and Ca-poor clay deposits) and firing strategies (e.g., oxidising atmosphere) for the manufacture of the coarse ware jars.

Second, the results in this study indicate that most jars (*N* = 27) from *Norba* were imported either from the Alban Hills or from the Tiber Valley, north of Rome, throughout the Middle and Late Republican eras. This is taken to suggest that *Norba* was integrated in trade networks that were prevalent in Southern Lazio during that time. In comparison, few coarse ware jars (*N* = 5) seem to have been produced within the region (e.g. *Satricum, Forum Appii*), or even locally at *Norba*. There seems to have been a change in these regional/local trade networks between the Middle and Late Republican eras. More specifically, some jars from *Norba* are compositionally similar to the repertoire from *Satricum*, indicating the marketing of coarse ware between *Satricum* and *Norba* during the Middle Republican era. Pottery production at *Satricum* is known to have ceased after the fourth century BCE, after which time the Inner plain of the Pontine Region and the surroundings of *Norba* developed with numerous farms, villa domains, roadside settlements, and workshops where ceramic building materials and fine ware were produced. The results in this study tentatively suggest that (one of) these workshops in the Inner plain or near *Norba* also produced coarse ware during the Late Republican era.

## Data Availability

All scientific data are held by the corresponding author and can be accessed for comparative purposes with prior arrangement.
